# A Case of a Figure of Eight Atrial Tachycardia after a Pulmonary Vein Antrum Isolation of Atrial Fibrillation

**DOI:** 10.31662/jmaj.2020-0018

**Published:** 2020-06-19

**Authors:** Yoshibumi Antoku, Masao Takemoto, Fuminaga Suetsugu, Takuya Tsuchihashi

**Affiliations:** 1 Cardiology, Cardiovascular Center, Steel Memorial Yawata Hospital, Kitakyushu, Japan; 2 Cardiology and Internal Medicine, Suetsugu Clinic, Kitakyushu, Japan

**Keywords:** atrial fibrillation, atrial tachycardia, catheter ablation, figure of eight, high-density mapping, pulmonary vein isolation

## Abstract

A 66-year-old female, whom received a pulmonary vein (PV) isolation (PVAI) with linear ablation of the carina lines between the superior and inferior PVs of both the right and left PVs for atrial fibrillation (AF), was admitted to receive a radiofrequency catheter ablation (RFCA) of symptomatic drug-refractory atrial tachycardia (AT). The EnSite^TM^ analysis by the Advisor^TM^ HD Grid catheter during the AT could easily detect that the carina between the right superior and inferior PVs exhibited a low voltage area (< 0.5 mV), in addition to the fact that the electrical activation turned around the right PVs in a figure 8, even though mapping was performed during AT. This AT was steadily terminated, after commencing the radiofrequency energy delivery to the carina of the right PVs.

## Introduction

The number of patients with atrial fibrillation (AF) is increasing, and radiofrequency catheter ablation (RFCA) of AF proved to be a useful strategy worldwide^(1), (2)^. However, the pulmonary vein (PV) antrum isolation (PVAI) for the treatment of AF rarely induces atrial tachycardias (ATs)^(3)^ and is well-known regarding this matter. Here, we report a case of a figure of eight ATs, which are comparably uncommon after a PVAI for AF^(4)^, that was successfully treated by RFCA. 

## Case Report

**Figure 1.**
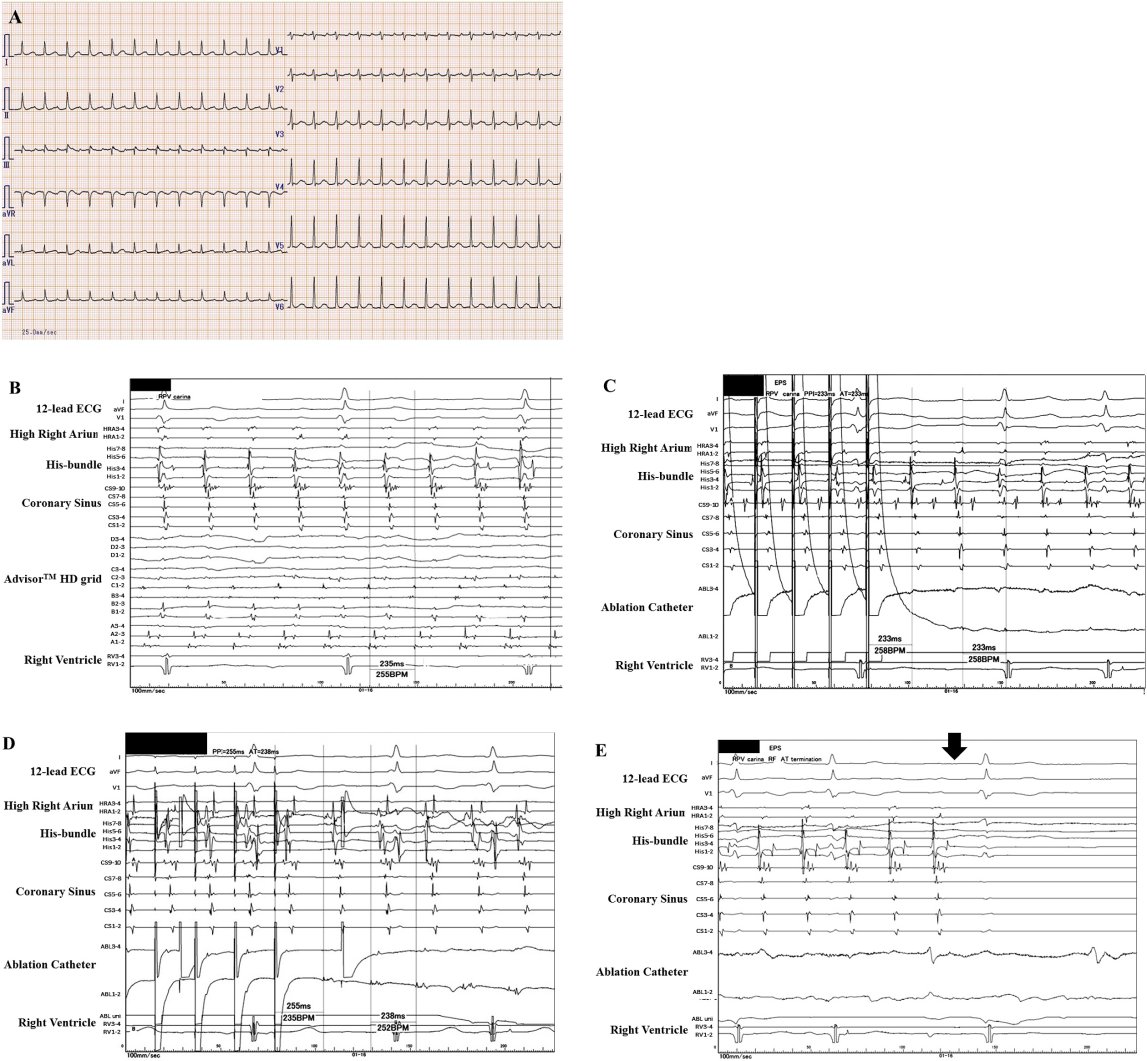
The 12-lead electrocardiograms on admission (A). An intra-cardiac electrocardiogram during atrial tachycardia (AT) (B–D). The EnSite^TM^ analysis by an Advisor^TM^ HD Grid catheter during AT demonstrated that the carina between the right veins (PVs) exhibited a low voltage (< 0.5 mV) (B). The concealed entrainment by the ablation catheter pacing positioned on the carina between the right PVs (C) and around both right PVs (D) were obtained. The post-pacing interval almost equaled the cycle-length of this AT (C, D). This AT steadily terminated (black arrow) within 2 seconds after commencing the radiofrequency energy delivery to the carina of the right PVs (E).

**Figure 2.**
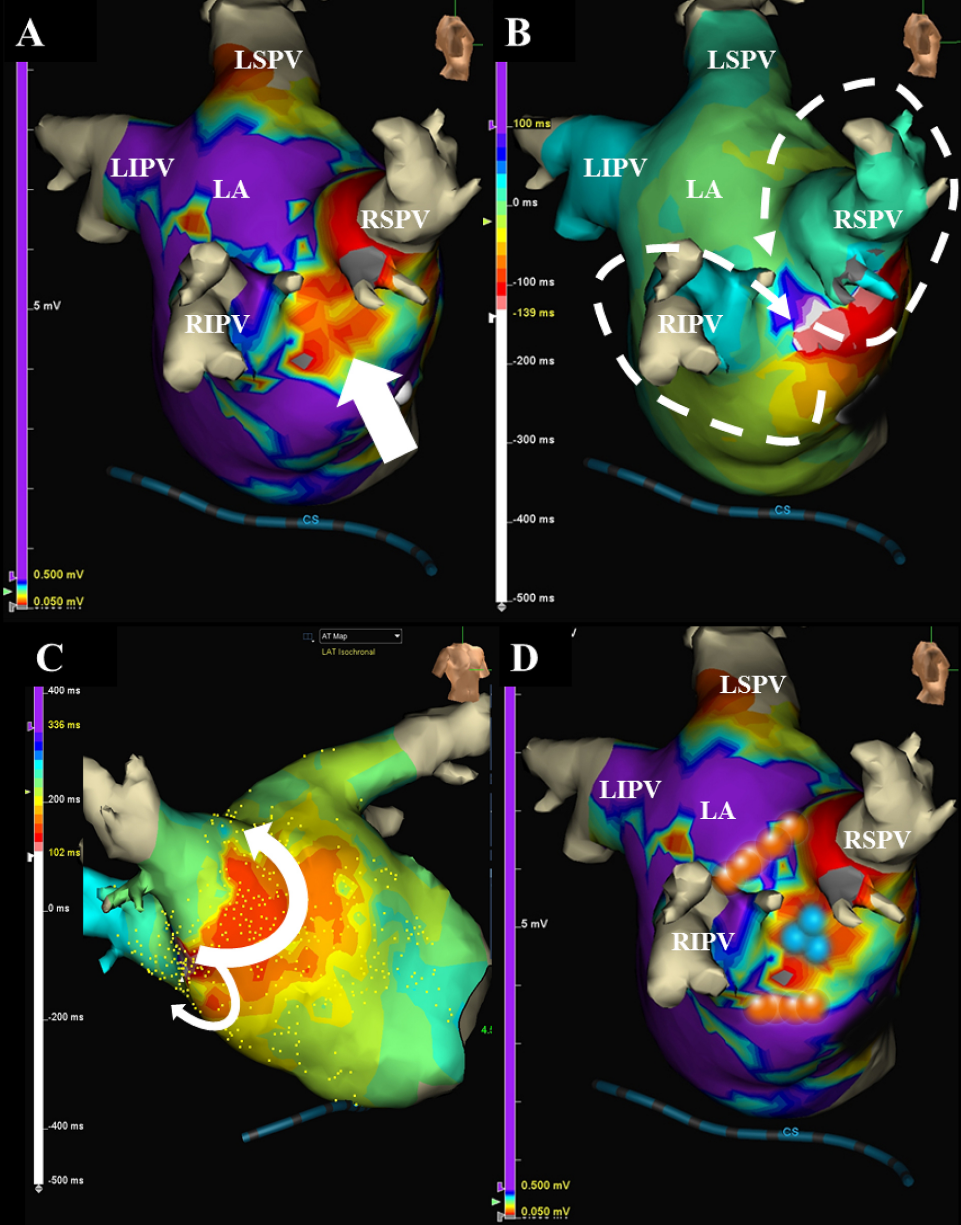
The EnSite^TM^ images viewed from the right demonstrate that the carina between the right superior and inferior pulmonary veins exhibits a low voltage (< 0.5 mV) (white arrow) (A). The electrical activation has turned around the right pulmonary veins in a figure of 8 circuit projected from right lateral view (white dotted lines) (B) and right anterior view (white lines) (C). The blue tags between the right superior and inferior pulmonary veins indicate the successful ablation points of the figure of 8 atrial tachycardia (D). The orange tags on the incomplete anterior and posterior lines of the previous pulmonary vein antrum isolation (PVAI) lines indicate the successful ablation points of the repeat PVAI (B).

A 66-year-old female, whom received an ipsilateral PVAI performed by a single continuous circular lesion by RFCA of the carina lines between the superior and inferior PVs of both the right and left PVs for paroxysmal AF 1-year prior, was admitted to receive a RFCA of intolerable symptomatic drug-refractory AT. This AT was a long RP’ type with a heart rate of 150 beat per minute (Figure 1A). Five-, 5-, and 4-French deflectable catheters were inserted and positioned into the coronary sinus, his-bundle and right ventricle, and high right atrium, respectively. After a trans-septal puncture, an EnSite^TM^ (Abbott, Plymouth, Minn, USA) analysis^(5)^ by an Advisor^TM^ HD Grid catheter (Abbott, Plymouth, Minn, USA) of both atria during the AT demonstrated that the carina between the right superior and inferior pulmonary veins (PVs) exhibited a low voltage (< 0.5 mV) (Figure 1B and white arrow in Figure 2A). Moreover, the electrical activation turned around the right PVs in a figure 8 circuit (Figure 2B, C) and Supplementary Material 2), and concealed entrainment was obtained at the carina (Figure 1C and Supplementary Material 1) and around both right PVs(Figure 1D). Thus, this AT was diagnosed as a macro-reentrant figure 8 AT including the carina between the right PVs as a slow conduction zone. After commencing the radiofrequency energy delivery to the carina of the right PVs (blue tags in Figure 2D), this AT was steadily terminated within 2 seconds (Figure 1E). Additional radiofrequency energy deliveries were performed, to complete the anterior and posterior lines of the previous incomplete PVAI lines (orange tags in Figure 2B). Thereafter, no atrial tachyarrhythmias, including AT and AF, could be completely induced by programmed stimulation of the atria. The patient remained well without any symptoms for 1 year after the RFCA.

## Discussion

Although the PVAI proved to be a useful strategy for RFCA of AF worldwide^(1), (2)^, the PVAI rarely induces ATs, and its incidence has been reported to be 4%^(3)^. Researchers reported that the mean period when an AT was documented after the initial PVAI is about 47 weeks, and about 60% of them are macro-reentrant tachycardias^(3)^. This present case may be a typical case because this AT was documented about 50 weeks after the initial PVAI and its mechanism(s) was macro-reentry. However, macro-reentrant figure 8 ATs are comparably uncommon after RFCA of AF^(4)^. The initial carina ablation between the right PVs was thought to be incomplete and created a slow conduction area, the so-called isthmus area (white arrow in Figure 2A). The previous anterior and posterior lines of the PVAI lines might also have been thought to be incomplete or may have caused re-conduction in that 1st year. Finally, re-entrant circuits around both the superior and inferior PVs were constituted (Figure 2B). The EnSite^TM^ analysis^(5)^ by the Advisor^TM^ HD Grid catheter of the left atria during the AT could easily detect that the carina between the right superior and inferior PVs exhibited a low voltage area (< 0.5 mV) (Figure 2A) and that the electrical activation turned around the right PVs in a figure 8 (Figure 2B, C), even though mapping was performed during AT. The high-density (HD) mapping catheter, Advisor^TM^ HD Grid, is the only directional HD mapping catheter that can not only identify the local electrical signals but more importantly can capture the direction of the wave front propagation, especially in low voltage zones^(6)^. In this present case, the HD mapping using an Advisor^TM^ HD Grid was able to show clear fractionated signals in the isthmus area of this figure 8 AT. As researchers reported that the acute success rate of ATs after an initial PVAI is about 80%^(3)^, this AT was steadily terminated within 2 seconds (Figure 1D) after commencing the radiofrequency energy delivery to the carina of the right PVs (blue tags in Figure 2D).

## Article Information

### Conflicts of Interest

None

### Acknowledgement

We thank Mr. Kensuke Kawasaki, Tomomi Hatae, Ryo Okada, Shu Takata, and Tsutomu Yoshinaga for their technical assistance with the electrophysiological study in the cardiac catheterization laboratory and Mr. John Martin for his linguistic assistance with this paper.

### Author Contributions

Drs. Antoku Y. and Takemoto M. performed radiofrequency catheter ablation. Dr. Suetsugu F. was in charge of out-patients. Dr. Tsuchihashi T. was in charge of the patient in our hospital.

### Informed Consent

The written consent was obtained from patients to publish the information.

### Approval by Institutional Review Board (IRB)

Not applicable.

## Supplement

Supplementary Material 1 The enlarged view of Figure 1C. The very small potential on ABL 1-2 after pacing could be seen (blue arrow).

Supplementary Material 2 The electrical activation turned around the right pulmonary veins in a figure of 8 circuit.
