# Supplementary figures and images for: A Case of a Figure of Eight Atrial Tachycardia after a Pulmonary Vein Antrum Isolation of Atrial Fibrillation
Source: JMA J. 2020 Jun 19;3(3):280–3. doi: 10.31662/jmaj.2020-0018 (PMC7590383; doi:10.31662/jmaj.2020-0018)

Supplemental file 1

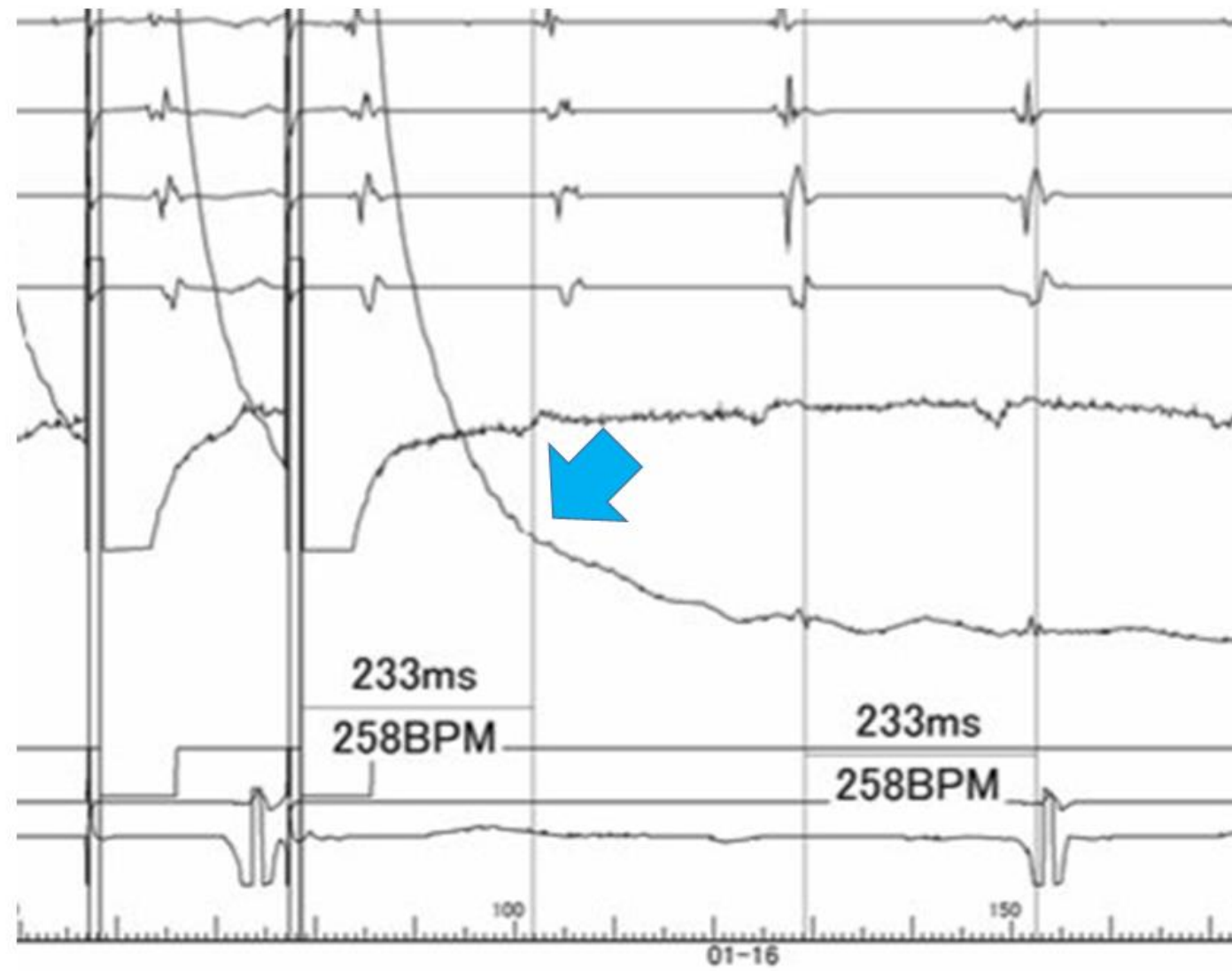

Supplement: Supplementary Material 1 — The enlarged view of Figure 1C. The very small potential on ABL 1-2 after pacing could be seen (blue arrow). [file 2433-3298-3-3-0280-s001.pdf]
